# Helping Adolescents with Greater Psychosocial Needs: Evaluation of a Positive Youth Development Program

**DOI:** 10.1100/tsw.2008.85

**Published:** 2008-06-13

**Authors:** Daniel T. L. Shek, Rachel C. F. Sun

**Affiliations:** ^1^ Centre for Quality of Life, Hong Kong Institute of Asia-Pacific Studies, The Chinese University of Hong Kong, Hong Kong; ^2^ Kiang Wu Nursing College of Macau, Macau; ^3^ Social Welfare Practice and Research Centre, The Chinese University of Hong Kong, Hong Kong

**Keywords:** secondary prevention, subjective outcome evaluation, volunteer training and services, adventure-based counseling

## Abstract

The Tier 2 Program of the Project P.A.T.H.S. (Positive Adolescent Training through Holistic Social Programmes), designed and implemented primarily by school social workers, attempts to help adolescents with greater psychosocial needs. After completion of the Tier 2 Program in the Full Implementation Phase (2006/07 school year), 10,255 Secondary 1 students in 207 schools responded to the Subjective Outcome Evaluation Form (Form C) to assess their views of the program, instructors, and perceived effectiveness of the program. Results showed that high proportions of the respondents had positive perceptions of the program and the instructors, and roughly four-fifths of the respondents regarded the program as helpful to them. Pearson correlation analyses showed that perceptions of the program and instructors were significantly correlated with perceived effectiveness of the program. Participants who joined volunteer training activities generally had higher global subjective outcome evaluation scores than did participants who attended programs without volunteer training activities.

### INTRODUCTION

The Project P.A.T.H.S. (Positive Adolescent Training through Holistic Social Programmes) is a positive youth development program that attempts to promote positive youth development in junior secondary school students in Hong Kong[1,2]. There are two tiers of programs (Tier 1 and Tier 2) in this project. The Tier 1 Program is a universal positive youth development program in which students in Secondary 1 to 3 take part. There are 20 h of training in both core and elective programs each school year for each grade. Based on different evaluation strategies, including objective outcome evaluation[3], subjective outcome evaluation[4], process evaluation[5], qualitative evaluation[6], and interim evaluation[7], there is evidence supporting the effectiveness of the Tier 1 Program[8].

Besides building up psychosocial competencies in adolescents via the Tier 1 Program, students with greater psychosocial needs are helped via the Tier 2 Program. Because research findings suggest that roughly one-fifth of adolescents need more help, the Tier 2 Program is provided for at least one-fifth of the students who display greater psychosocial needs at each grade (i.e., selective prevention). The researchers deliberately avoided using the term "at risk" because the term is very stigmatizing in the Chinese culture, and it deters parents and students from joining the related programs.

For students who are identified as having special needs, they and/or their families receive services based on the Tier 2 Program. To create more flexibility for the workers with reference to the specific needs of the students in the unique school context, the NGO applicants have the choice of designing appropriate programs that target the needs of the students with reference to the 15 positive youth development constructs maintained in the project, as well as goals and objectives covered in this project[9]. Several non-mutually exclusive examples for the Tier 2 Program include: (a) mentorship programs involving the alumni of the schools, (b) mental health promotion programs, (c) adventure-based counseling, (d) parenting programs, (e) service learning programs, and (f) resilience enhancement programs.

In a pioneering study of the subjective outcome evaluation of the Tier 2 Program, Shek et al.[10] examined subjective outcome evaluation in 2,173 students in 52 schools after they completed the Tier 2 Program. Based on the consolidated reports submitted by the agencies to the funding body, the research team aggregated the consolidated data to form a "reconstructed" overall profile on the perceptions of the program participants. Four major types of program were identified, including programs based on the adventure-based counseling approach (N = 8), programs concentrated on volunteer training and services (N = 7), programs incorporating both adventure-based counseling and volunteer training elements (N = 30), and other programs with different foci (N = 7). Results showed that high proportions of the respondents had positive perceptions of the programs and the instructors, and roughly four-fifths of the respondents regarded the program as helpful to them.

Although the findings of Shek et al.[10] are pioneering in nature, there are several limitations of this study. First, the findings were based on the data collected in the Experimental Implementation Phase only. As such, it would be illuminating to look at this issue with reference to the Full Implementation Phase. Second, the study was primarily descriptive in nature, which did not examine the relationship between qualities of the program and program implementers and perceived effectiveness. Finally, as the number of participating schools in the Experimental Implementation Phase was small, it was difficult to address the question of whether participants of different Tier 2 Programs had different subjective outcome evaluation ratings.

There are views that suggest that the quality of the program and the workers who implement the program are important determinants of program effectiveness. Nation et al.[11] pointed out that a comprehensive program and well-trained program implementers are important ingredients of an effective program. Weissberg[12] also pointed out that a well-designed program and high-quality program implementers were commonly found in effective school-based social-emotional learning programs. There are research findings that also show that qualities of the program and program implementers are related to program outcomes: Harachi et al.[13] showed that instructional strategies (proactive classroom management, cooperative learning methods, strategies to enhance student motivation, student involvement and participation, reading strategies, and interpersonal and problem-solving skills training) were related to student social competencies; Tobler et al.[14] showed that programs with high peer interaction were more effective than programs with low peer interaction and that the delivery method instead of the program content determined the success of the program. According to these research findings, it was hypothesized that perceived program characteristics and program implementers would be positively related to perceived helpfulness of the program.

Regarding the different modes of Tier 2 Programs, although the previous study showed that adventure-based counseling and volunteer training and services were the two major modes involved, comparative analyses were not meaningful because of the small sample size. As far as adventure-based counseling is concerned, it is an approach that attempts to integrate adventure, wilderness, and experiential learning, as well as individual and group counseling[15,16]. According to this approach, when an adolescent with a disequilibrium in personal development is put into an environment that is strange and requires cooperation (i.e., adventure environment), the tasks designed to provide adventure experiences will lead to transformation in the participant, including changes in self-confidence and self-understanding, as well as cooperation with others[17,18]. According to Schoel et al.[19], adventure-based counseling promotes life skills in the participants, including communication skills, cooperation, and decision-making and problem-solving skills.

There are research findings that support the effectiveness of wilderness therapy. In a review of 19 studies of the effectiveness of adventure-based counseling, Moote and Wodarski[20] showed that 16 of the 19 studies reported some positive effects for the participants, including enhanced self-esteem; self-concept; cooperative behavior; and physical, social, and intellectual growth. They also concluded that "for social workers who provide direct services to adolescents in various settings, adventure-based counseling may be a viable alternative to traditional approaches" (p. 161–162).

The second major mode of Tier 2 Programs is closely related to volunteering training and services. According to Clary et al.[21], there are six functions of volunteering. These include enhanced understanding of the world through volunteering (knowledge function); expression of values via volunteering (value expressive function); avoidance of personal issues or undesirable truths about the self via volunteering (ego defensive function); enhancement of self-esteem, competence, and mood (self-enhancement function); facilitation of career and development of a better resume (utilitarian function); and social companionship and socializing with other volunteers (interpersonal function). There are research findings that also show that volunteers believed in several benefits of volunteering, including knowledge and skill acquisition, enhancement in occupational and educational opportunities, and social belongingness among peers[22,23,24]. In addition, there are research findings that show that volunteerism is associated with a lower level of anticipated distress and negative emotion[25,26]. Based on these findings, it would be expected that volunteer training and services would promote positive youth development.

This paper presents and discusses the findings of an outcome evaluation of the Tier 2 Program implemented in the Full Implementation Phase of the project in 2006/07 based on the perspective of the participants. Besides describing the profile of responses based on subjective outcome evaluation, the relationships between perceived quality of the program and program implementers and perceived program effectiveness were examined. Furthermore, whether participants who joined different programs differ in their evaluation of the program will be examined. It is noteworthy that although it is very common for social work agencies to design programs for adolescents with greater psychosocial needs (e.g., adventure-based counseling, volunteer training program), systematic evaluation and documentation of program evaluation have been rarely found in the local social work literature[3].

### METHODS

#### Participants and Procedures

A total of 207 schools joined the Full Implementation Phase of the project in 2006/07. In these schools, there were 13,194 participants involved in the Tier 2 Program. Among them, 12,092 students were identified by teachers, parents, and/or self-administered questionnaires as having greater psychosocial needs, and they were invited to join the Tier 2 Program. In addition, 1,102 participants who are the parents and teachers of those identified students also participated as part of the Tier 2 Program. The mean number of participants joining the Tier 2 Program per school was 63.74 (range: 14–308 participants). The average number of sessions provided per school (normally 1.5–3 h per session) was 22.91 (range: 6–62 sessions).

The participants were invited to respond to a subjective outcome evaluation questionnaire, usually immediately after completion of the Tier 2 Program. A total of 10,255 participants (mean = 49.54 participants per school, range: 6–294) responded to the Subjective Outcome Evaluation Form (Form C) developed by the research team[27]. The overall response rate was 77.72%. There are three plausible reasons for the moderately low response rate: (1) participants withdrew early from Tier 2 Program, (2) participants were absent from the last session and did not complete the evaluation form, or (3) some schools did not invite the adult participants to respond to the evaluation form.

On the day of data collection, the purpose of the evaluation was explained and the principle of confidentiality was repeatedly emphasized to the participants. The participants were asked to indicate their wish if they did not want to respond to the evaluation questionnaire (i.e., "passive" informed consent was obtained). All participants responded to all scales in the evaluation form in a self-administration format and in an anonymous manner. Adequate time was provided for the participants to complete the questionnaire. To facilitate the program evaluation, the research team developed an evaluation manual with standardized instructions for collecting the subjective outcome evaluation data[27]. In addition, adequate training was provided to the social workers during 20-h training workshops on how to collect and analyze the data collected using Form C.

### Instruments

The Subjective Outcome Evaluation Form (Form C) designed by Shek and Siu[27] aims to measure the perceptions of the Tier 2 Program. There are seven parts in this evaluation form:

- Participants' perceptions of the program, such as program design, quality of service, appropriateness of the program, and interaction among the participants (8 items).
- Participants' perceptions of the workers, such as the preparation of the workers, professional attitude and knowledge, and interaction with the participants (8 items).
- Participants' perception of the effectiveness of the program, such as resilience and overall personal development (8 items).
- Things that the participants appreciated most (open-ended question).
- Opinion about the workers (open-ended question).
- Things that the participants learned from the program (open-ended question).
- Areas that require improvement (open-ended question).

After collecting the data, the social work service providers were requested to input the data into an EXCEL file developed by the research team that would automatically compute the frequencies and percentages associated with the different ratings for an item. When the providers submitted the reports, they were also requested to submit the soft copy of the consolidated data sheets. The data from all service providers were then aggregated to "reconstruct" the overall profile. Since some amendments were made during the program implementation (e.g., program cancellation due to insufficient participants), the delivered program content was reported again in the program report.

Since the program contents of the Tier 2 Programs were designed according to the various needs and the contextual situations of different schools, it is hard to compare their results directly. Therefore, an analysis on the program contents was conducted to identify the major program elements of the Tier 2 Programs for all participating schools. Two trained research assistants (both with the Bachelor degree in Social Work and have certain front-line social work experiences for adolescents) categorized the program contents delivered by the social workers as indicated in the program reports submitted to the research team. The criteria were strictly set to categorize a program according to the program proposal or revised proposal, together with the actual activities. By randomly selecting 10 cases by each trained research assistant, intra-rater reliability was 100 and 100%, respectively. Furthermore, when two other raters were invited to code 10 randomly selected cases, an inter-rater reliability of 100% was found.

## RESULTS

Reliability analysis with the schools as the unit of analyses showed that Form C was internally consistent: eight items related to the program (alpha = 0.98, mean inter-item correlation = 0.88), eight items related to the workers (alpha = 0.99, mean inter-item correlation = 0.90), eight items related to the effectiveness

(alpha = 0.99, mean inter-item correlation = 0.90), and 24 items based on the whole Form C (alpha = 0.99, mean inter-item correlation = 0.84).

Results of the content analyses showed that three major types of program elements were identified, namely, the adventure-based counseling approach, volunteer training and services, and others. First, a program would be classified as containing the adventure-based activities and counseling (ABC) elements if and only if the espoused theory was matched with the theory-in-action[28]. Second, programs would be classified as volunteer training and services if they were related to program planning skills and organizing volunteer services. The most popular volunteer services found in the Tier 2 Program were visiting the elderly and services for mentally challenged people. Third, programs without elements of ABC or volunteer training and services were classified as “others”, and this category included groups that promote learning skills, a social skills training program, and a therapeutic group with Narrative Therapy Approach.

As shown in Table 1, the Tier 2 Programs were further categorized into four types by the main types of program elements used or program approaches adopted. They are (1) the ABC approach together with volunteer training and services (Type A, 53.14%), (2) ABC only (Type B, 27.54%), (3) volunteer training and services only (Type C, 11.59%), and (4) other approaches (Type D, 7.73%). An analysis of the programs delivered showed that the ABC approach and volunteer training and services were the two major program elements adopted for the Tier 2 Programs, and over 90% of all programs delivered by the 207 secondary schools contained at least one of these two elements. The mean overall effectiveness of all Tier 2 Programs ranged from 4.04 to 5.13 on a six-point scale towards the positive side. The average number of positive youth development constructs indicated in the reports for each type of programs ranged from 3 to 9 and the mode was 3.

**TABLE 1**
**Summary of Program Characteristics and Effectiveness**

| Main Program Approach | Clientele* | Average No. of Participants | Average Program Attendance (%) | Average No. of Program Aims Indicated in the Reports | Average No. of Constructs Indicated in the Reports | Mean of Overall Effectiveness |
|---|---|---|---|---|---|---|
| ABC approach and volunteer training and services (Type A) (N = 110) | A (N = 79) | 50.15 | 82.58 | 1.97 | 5.67 | 4.54 |
| | B (N = 23) | 77.35 | 82.33 | 1.83 | 7.96 | 4.35 |
| | C (N = 1) | 47.00 | 92.00 | 2.00 | 5.00 | 5.13 |
| | D (N = 7) | 111.29 | 86.39 | 2.00 | 6.57 | 4.76 |
| ABC approach only (Type B) (N = 57) | A (N = 41) | 52.63 | 81.25 | 2.10 | 6.24 | 4.45 |
| | B (N = 7) | 52.29 | 76.02 | 2.00 | 5.43 | 4.60 |
| | C (N = 1) | 80.00 | 88.30 | 1.00 | 5.00 | 4.36 |
| | D (N = 8) | 137.63 | 81.25 | 2.50 | 6.38 | 4.04 |
| Volunteer training and services only (Type C) (N = 24) | A (N = 21) | 48.62 | 82.64 | 2.05 | 6.14 | 4.41 |
| | B (N = 1) | 290.00 | 78.20 | 1.00 | 8.00 | 4.84 |
| | D (N = 2) | 70.00 | 84.69 | 3.50 | 9.00 | 4.15 |
| Other approaches (Type D) (N = 16) | A (N = 11) | 57.91 | 88.24 | 2.18 | 5.00 | 4.42 |
| | B (N = 3) | 128.67 | 90.47 | 2.00 | 3.00 | 4.26 |
| | D (N = 2) | 147.50 | 92.25 | 1.50 | 5.50 | 4.16 |

* A, only students involved; B, students and parents involved; C, students and teachers involved; D, students, parents, and teachers involved.

Based on the quantitative data derived from the closed-ended questions, there are several observations. First, over 80% of the respondents perceived the Tier 2 Program in a positive manner (Table 2). For example, 85.42% of the participants indicated that they had much interaction with other participants; 85.34% indicated that they were satisfied with the service. Second, a very high proportion of the participants had positive evaluation of the workers implementing the program (Table 3). For example, nearly 90% of the participants indicated that the worker(s) were well prepared for the program, and about 88% indicated that the worker(s) had professional knowledge and good attitudes. Third, as shown in Table 4, roughly four-fifths of the participants perceived that the program had induced positive changes in them. For example, they learned how to help themselves (85.57%) and solve problems (85.4%) through participating in the program.

**TABLE 2**
**Comparison of the Positive Views Towards the Tier 2 Program Among Different Program Approaches**

| | ABC Approach and Volunteer Training and Services[1] | | ABC Approach[2] | | Volunteer Training and Services[3] | | Others[4] | | Overall | |
|---|---|---|---|---|---|---|---|---|---|---|
| | N (Total Response) | % | N (Total Response) | % | N (Total Response) | % | N (Total Response) | % | N (Total Response) | % |
| The activities were carefully planned. | 3,941 (4,610) | 85.49 | 2,712 (3,350) | 80.96 | 833 (1,019) | 81.75 | 999 (1,223) | 81.68 | 8,485 (10,202) | 83.17 |
| The quality of the service was high. | 3,900 (4,609) | 84.62 | 2,611 (3,341) | 78.15 | 828 (1,019) | 81.26 | 975 (1,223) | 79.72 | 8,314 (10,192) | 81.57 |
| The service provided could meet the participants' needs. | 3,914 (4,599) | 85.11 | 2,672 (3,340) | 80.00 | 828 (1,021) | 81.10 | 979 (1,217) | 80.44 | 8,393 (10,177) | 82.47 |
| The service delivered could achieve the planned objectives. | 3,969 (4,598) | 86.32 | 2,746 (3,337) | 82.29 | 854 (1,018) | 83.89 | 995 (1,221) | 81.49 | 8,564 (10,174) | 84.18 |
| Participants could get the service they wanted. | 3,839 (4,589) | 83.66 | 2,567 (3,338) | 76.90 | 817 (1,016) | 80.41 | 950 (1,217) | 78.06 | 8,173 (10,160) | 80.44 |
| Participants had much interaction with other participants. | 3,976 (4,599) | 86.45 | 2,849 (3,334) | 85.45 | 827 (1,013) | 81.64 | 1,028 (1,216) | 84.54 | 8,680 (10,162) | 85.42 |
| Participants would recommend others who have similar needs to participate in the program. | 3,724 (4,595) | 81.04 | 2,582 (3,334) | 77.44 | 794 (1,017) | 78.07 | 924 (1,219) | 75.80 | 8,024 (10,165) | 78.94 |
| On the whole, participants were satisfied with the service. | 4,039 (4,595) | 87.90 | 2,751 (3,340) | 82.37 | 854 (1,017) | 83.97 | 1,038 (1,222) | 84.94 | 8,682 (10,174) | 85.34 |

*Note:* [1]The program contents related to both the ABC approach and volunteer training and services were indicated in the Tier 2 program reports. [2]The program contents related to the ABC approach were indicated in the Tier 2 program reports. [3]The program contents related to volunteer training and services were indicated in the Tier 2 program reports. [4]Except the ABC approach and volunteer training and services, other program contents were indicated in the Tier 2 program reports.

**TABLE 3**
**Comparison of the Positive Views Towards the Tier 2 Program Workers Among Different Program Approaches**

| | ABC Approach and Volunteer Training and Services[1] | | ABC Approach[2] | | Volunteer Training and Services[3] | | Others[4] | | Overall | |
|---|---|---|---|---|---|---|---|---|---|---|
| | N (Total Response) | % | N (Total Response) | % | N (Total Response) | % | N (Total Response) | % | N (Total Response) | % |
| The worker(s) had professional knowledge. | 4,102 (4,602) | 89.14 | 2,907 (3,341) | 87.01 | 895 (1,018) | 87.92 | 1,053 (1,220) | 86.31 | 8,957 (10,181) | 87.98 |
| The worker(s) demonstrated good working skills. | 4,073 (4,600) | 88.54 | 2,771 (3,335) | 83.09 | 889 (1,019) | 87.24 | 1,034 (1,220) | 84.75 | 8,767 (10,174) | 86.17 |
| The worker(s) were well prepared for the program. | 4,185 (4,602) | 90.94 | 2,922 (3,332) | 87.70 | 909 (1,017) | 89.38 | 1,072 (1,220) | 87.87 | 9,088 (10,171) | 89.35 |
| The worker(s) understood the needs of the participants. | 4,086 (4,597) | 88.88 | 2,730 (3,331) | 81.96 | 875 (1,017) | 86.04 | 1,029 (1,215) | 84.69 | 8,720 (10,160) | 85.83 |
| The worker(s) cared about the participants. | 4,140 (4,598) | 90.04 | 2,794 (3,330) | 83.90 | 898 (1,018) | 88.21 | 1,043 (1,217) | 85.70 | 8,875 (10,163) | 87.33 |
| The worker(s)' attitudes were very good. | 4,151 (4,593) | 90.38 | 2,797 (3,335) | 83.87 | 914 (1,012) | 90.32 | 1,060 (1,215) | 87.24 | 8,922 (10,155) | 87.86 |
| The worker(s) had much interaction with participants. | 3,954 (4,594) | 86.07 | 2,642 (3,333) | 79.27 | 855 (1,015) | 84.24 | 986 (1,218) | 80.95 | 8,437 (10,160) | 83.04 |
| On the whole, participants were satisfied with the worker(s). | 4,176 (4,598) | 90.82 | 2,851 (3,335) | 85.49 | 9,10 (1,018) | 89.39 | 1,066 (1,219) | 87.45 | 9,003 (10,170) | 88.53 |

*Note:* [1]The program contents related to both the ABC approach and volunteer training and services were indicated in the Tier 2 program reports. [2]The program contents related to the ABC approach were indicated in the Tier 2 program reports. [3]The program contents related to volunteer training and services were indicated in the Tier 2 program reports. [4]Except the ABC approach and volunteer training and services, other program contents were indicated in the Tier 2 program reports.

Furthermore, Table 5 shows that various domains of the subjective outcome evaluation were inter-related, i.e., the overall views about the program and the overall views about the instructors were significantly correlated with each other, and both of them were positively correlated with all eight aspects of the perceived effectiveness of the program. The hypotheses outlined in the earlier part of the paper were confirmed.

Comparisons between different program approaches were carried out, by conducting two sets of analyses. The first set of analyses addressed the question of whether programs with and without ABC elements differed in the subjective outcome evaluation ratings. As shown in Table 6, analyses showed that the two types of programs did not differ. The second set of analyses addressed the issue of whether programs with and without volunteer training activities differ in subjective outcome evaluation ratings. Although the participants in both groups had similar views about the program and program benefits, those attending the volunteer training and services–related programs in the Tier 2 Program had significantly better views about their instructors and the overall program (Table 6).

**TABLE 4**
**Comparison of the Positive Views Towards the Tier 2 Program Effectiveness Among Different Program Approaches**

| | ABC Approach and Volunteer Training and Services[1] | | ABC Approach[2] | | Volunteer Training and Services[3] | | Others[4] | | Overall | |
|---|---|---|---|---|---|---|---|---|---|---|
| | N (Total Response) | % | N (Total Response) | % | N (Total Response) | % | N (Total Response) | % | N (Total Response) | % |
| The service helped participants a lot. | 3,846 (4,578) | 84.01 | 2,699 (3,320) | 81.30 | 824 (1,010) | 81.58 | 970 (1,210) | 80.17 | 8,339 (10,118) | 82.42 |
| The service enhanced participants' growth. | 3,941 (4,576) | 86.12 | 2,743 (3,319) | 82.65 | 840 (1,009) | 83.25 | 984 (1,208) | 81.46 | 8,508 (10,112) | 84.14 |
| In the future, participants would receive similar service(s) if needed. | 3,793 (4,571) | 82.98 | 2,559 (3,321) | 77.06 | 809 (1,007) | 80.34 | 946 (1,207) | 78.38 | 8,107 (10,106) | 80.22 |
| Participants learned how to help themselves through participating in the program. | 3,965 (4,569) | 86.78 | 2,822 (3,317) | 85.08 | 846 (1,006) | 84.10 | 1,007 (1,205) | 83.57 | 8,640 (10,097) | 85.57 |
| Participants had positive change(s) after joining the program. | 3,898 (4,560) | 85.48 | 2,742 (3,315) | 82.71 | 842 (1,007) | 83.61 | 989 (1,207) | 81.94 | 8,471 (10,089) | 83.96 |
| Participants learned how to solve their problems through participating in the program. | 3,952 (4,548) | 86.90 | 2,789 (3,302) | 84.46 | 844 (1,003) | 84.15 | 999 (1,199) | 83.32 | 8,584 (10,052) | 85.40 |
| Participants' behavior became better after joining this program. | 3,749 (4,572) | 82.00 | 2,554 (3,312) | 77.11 | 800 (1,006) | 79.52 | 909 (1,206) | 75.37 | 8,012 (10,096) | 79.36 |
| Those who knew the participants agreed that this program induced positive changes in them. | 3,696 (4,563) | 81.00 | 2,495 (3,312) | 75.33 | 800 (1,006) | 79.52 | 914 (1,207) | 75.72 | 7,905 (10,088) | 78.36 |

*Note:* [1]The program contents related to both the ABC approach and volunteer training and services were indicated in the Tier 2 program reports. [2]The program contents related to the ABC approach were indicated in the Tier 2 program reports. [3]The program contents related to volunteer training and services were indicated in the Tier 2 program reports. [4]Except the ABC approach and volunteer training and services, other program contents were indicated in the Tier 2 program reports.

### DISCUSSION

Several observations can be highlighted from the present study. First, results showed that the various measures derived from Form C were internally consistent. With reference to the comments of Royse[29] that the lack of standardized assessment tools for conducting client satisfaction surveys also introduces biases for the client satisfaction approach, and that the use of assessment tools with known reliability and validity would "eliminate many of the problems found in hastily designed questionnaires" (p. 265), the present study is an interesting addition to the literature.

**TABLE 5**
**Intercorrelations Among Domains in Subjective Outcome Evaluation**

| Variables/Items | Views About Program | Views About Instructors |
|---|---|---|
| Views about program | 1 | 0.92* |
| Views about instructors | 0.92* | 1 |
| Perceived effectiveness of the program | | |
| The service helped participants a lot. | 0.92* | 0.87* |
| The service enhanced participants' growth. | 0.89* | 0.84* |
| In the future, participants would receive similar service(s) if needed. | 0.90* | 0.85* |
| Participants learned how to help themselves through participating in the program. | 0.89* | 0.85* |
| Participants had positive change(s) after joining the program. | 0.90* | 0.86* |
| Participants learned how to solve their problems through participating in the program. | 0.88* | 0.84* |
| Participants' behavior became better after joining this program. | 0.85* | 0.80* |
| Those who knew the participants agreed that this program induced positive changes in them. | 0.81* | 0.76* |

*Note:* A Bonferroni adjustment to the alpha level (17 pairs of correlations) was used.

*$p < 0.003$.

**TABLE 6**
**Comparison of the Views About the Tier 2 Program Among Different Program Approaches**

| Variable | Program Approach | Mean (Standard Deviation) | F-value |
|---|---|---|---|
| Comparison Between ABC Approach–Related Programs and Others | | | |
| Views about program | 1 | 4.57 (0.40) | 1.87 n.s. |
| | 2 | 4.47 (0.39) | |
| Views about instructors | 1 | 4.77 (0.38) | 0.45 n.s. |
| | 2 | 4.73 (0.36) | |
| Perceived effectiveness of the program | 1 | 4.56 (0.41) | 1.11 n.s. |
| | 2 | 4.48 (0.39) | |
| Global views | 1 | 4.63 (0.38) | 1.15 n.s. |
| | 2 | 4.56 (0.37) | |
| Comparison Between Volunteer Training and Services–Related Programs and Others | | | |
| Views about program | 3 | 4.59 (0.40) | 3.62 n.s. |
| | 4 | 4.48 (0.38) | |
| Views about instructors | 3 | 4.81 (0.36) | 4.91* |
| | 4 | 4.69 (0.40) | |
| Perceived effectiveness of the program | 3 | 4.58 (0.42) | 3.42 n.s. |
| | 4 | 4.47 (0.37) | |
| Global views | 3 | 4.66 (0.38) | 4.21* |
| | 4 | 4.55 (0.37) | |

*Note:* *$p < 0.05$; n.s. = nonsignificant. 1, ABC approach–related programs (Group A and Group B); 2, others (Group C & Group D); 3, volunteer training and services–related programs (Group A and Group C); 4, others (Group B and Group D).

Second, high proportions of the participants perceived the program in a positive light: most of the respondents had favorable perceptions of the program and workers implementing the program; roughly four-fifths of the respondents perceived the program to be beneficial to their own development. These observations generally suggest that the Tier 2 Program was perceived positively by the program participants and they felt that the program was helpful to them. These findings are also consistent with those emerging from the Experimental Implementation Phase[10] suggesting that the Tier 2 Program is beneficial to the program participants. Of course, these positive findings should be viewed together with the limitations of subjective outcome evaluation, such as demand characteristics and halo effect. If resources permit, studies attempting to examine the convergence of objective outcome evaluation findings and subjective outcome evaluation findings should be carried out.

Consistent with our prediction, participants' perceptions of the program were positively correlated with perceived benefits of the program. Similarly, participants' perceptions of the program implementers were positively correlated with perceived benefits of the program. These findings basically suggest that the quality of the program and program implementers are intimately related to perceived benefits of the program. As there are few studies that examine the correlates of program effectiveness based on a subjective outcome evaluation approach in the Chinese culture, the present findings can be regarded as pioneering additions to the literature[5].

Finally, the present findings give some insights on the differences in subjective outcome evaluation findings across different programs. There are two interesting observations deserving our attention. First, there were no differences between programs utilizing and not utilizing the ABC approach. Although the ABC approach has a long history in Hong Kong and has been formally adopted as the major program theory for a huge social intervention program entitled "The Understanding the Adolescent Project" (UAP) to combat the problems among students identified as adolescent-at-risk from 2001 to 2004 in Hong Kong, very few studies have systematically examined its effectiveness.

Second, participants who joined programs with volunteer training activities had higher global subjective outcome scores than did participants who joined programs without volunteer training activities. The positive evaluation findings based on the service learning programs are basically consistent with the literature that engagement in voluntary services is conducive to the positive development of adolescents. Nevertheless, as the present findings were "reconstructed" from the evaluation reports submitted by the agencies, the units of analyses were schools instead of individuals. As such, the power of the statistical analyses would become low and individual variations were lost in the process. Obviously, analyses based on data collected from individual participants should be attempted in future.

### ACKNOWLEDGMENTS

The preparation for this paper and the Project P.A.T.H.S. were financially supported by the Hong Kong Jockey Club Charities Trust.
